# Stakeholders’ Perspectives on Attrition in the Dental Therapy Profession in South Africa

**DOI:** 10.1002/puh2.70150

**Published:** 2025-10-22

**Authors:** Pumla Pamella Sodo, Yolanda Malele‐Kolisa, Aneesa Moolla, Tshakane Ralephenya, Veerasamy Yengopal, Simon Nemutandani, Sara Jewett

**Affiliations:** ^1^ Department of Family Medicine and Primary Care University of the Witwatersrand Johannesburg South Africa; ^2^ School of Oral Health Sciences University of the Witwatersrand Johannesburg South Africa; ^3^ Faculty of Dentistry University of the Western Cape Cape Town South Africa; ^4^ Faculty of Health Sciences School of Medicine University of Limpopo Polokwane South Africa; ^5^ School of Public Health University of the Witwatersrand Johannesburg South Africa

**Keywords:** career pathways, dental therapists, health policy, Herzberg's two‐factor theory, human resources for health, primary healthcare, professional identity, South Africa, workforce attrition, workforce retention

## Abstract

**Background:**

Dental therapists play a critical role in addressing oral health needs, especially in underserved areas. However, the South African health system faces workforce shortages and challenges retaining mid‐level oral health professionals. Understanding attrition from the perspective of stakeholders involved in training, policy and regulation is key to addressing systemic barriers and improving retention.

**Aim:**

This study aimed to explore the perspectives of key stakeholders on the factors contributing to attrition among dental therapists in South Africa, focusing on intrinsic and extrinsic influences as guided by Herzberg's two‐factor theory.

**Methods:**

Through a qualitative exploratory study design, in‐depth interviews were conducted with stakeholders to explore their perspectives on dental therapists’ attrition. They were recruited using snowball sampling. All interviews were audio recorded, transcribed verbatim, and coded in NVivo 12. A team of researchers applied thematic analysis to agree on themes and sub‐themes, guided by Hertzberg's ideas of intrinsic and extrinsic factors.

**Findings:**

Thirteen stakeholders participated in the study. Both extrinsic (policy implementation gaps, inadequate remuneration, limited recruitment opportunities and lack of professional identity) and intrinsic (limited career growth and unclear career pathways) factors were identified as key contributors to attrition. Despite the recognition of their essential role in primary healthcare (PHC), systemic challenges undermined professional satisfaction and retention.

**Conclusion:**

The study fulfils its aim by providing a nuanced understanding of attrition from the perspective of stakeholders. Addressing both systemic (extrinsic) and motivational (intrinsic) factors is critical to strengthening workforce sustainability. Policy reforms, structured career pathways and enhanced professional recognition are necessary to retain dental therapists and ensure their integration into South Africa's PHC vision.

## Graphical abstract

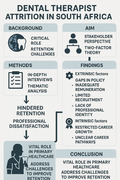

## 1 Introduction and Background

Attrition in public health professions, particularly among dental therapists, is a global concern driven by factors, such as poor remuneration, limited career progression, lack of recognition and job scarcity. In South Africa, this issue is compounded by systemic inequalities between the public and private healthcare sectors. Dental therapists, primarily serving in the underfunded public sector, face challenges, including geographic maldistribution and lack of access to further training Managers, regulators and other stakeholders, play a crucial role in ensuring the sustainability of professions through training, deployment and retention, which contribute to the improvement of the health status of the population. Globally, the production and retention of dental therapists has been a challenge throughout the years, with many dental therapists leaving the profession for reasons, such as poor remuneration, unclear career paths, lack of recognition and lack of jobs [1, 2, 3, 4, 5].

South Africa's healthcare system is divided into two tiers and is marked by significant inequality. The public sector, which is funded by the state, serves the majority of the population (approximately 71%). In contrast, the private sector, which relies mainly on individual contributions to medical aid schemes or health insurance, caters to about 27% of the population [6]. Although the public sector suffers from underfunding, the high cost of private healthcare remains unaffordable for most South Africans [6]. Dental therapists were introduced to operate largely within the public system, playing a preventive and curative role within the primary healthcare model.

Human resources for health (HRH) strategies aim to address these challenges by ensuring adequate training, recruitment and retention of healthcare workers. However, current policies fall short in supporting dental therapists, leading to high attrition rates. Understanding the root causes of this attrition requires input from health system leaders and managers, whose perspectives can reveal whether the problem lies in policy design or implementation.

The goals of HRH are centred around ensuring that a healthcare system has an adequate and competent workforce to meet the healthcare needs of its population, improve access to health services and achieve universal coverage. These goals typically include training, recruiting and retaining skilled healthcare professionals, including dental therapists, to address shortages and distribution gaps. Such issues have been identified in South Africa, with most dental therapists residing in only two out of nine provinces, and some provinces with less than 10 dental therapists [7]. HRH strategies also aim to improve the quality of healthcare services by providing ongoing training and professional development opportunities for healthcare workers. However, South African studies reported that dental therapists do not have access to further training within their profession, which has contributed to many dental therapists leaving their profession for other disciplines with clearer career prospects [3, 4].

To achieve these HRH goals, particularly within the dental therapy profession, it is essential to address the factors contributing to workforce attrition [8, 9, 10]. This requires both building on existing evidence and conducting new research to inform comprehensive and context‐specific mitigation strategies. Critically, the perspectives of those in key managerial and leadership roles within the health system must be included, as they are directly involved in the implementation and oversight of workforce policies. Their insights are vital to understanding whether attrition stems from shortcomings in policy design or from challenges in policy application [11]. Including these system‐level perspectives allows for a more nuanced understanding of structural and operational barriers affecting dental therapist retention. Such research can also inform the development of effective strategies to attract and retain dental therapists, thereby strengthening the health system's capacity to deliver high‐quality care, improve health outcomes and promote equitable access to healthcare services across communities.

Although there is a growing literature on the attrition of dental therapists in South Africa, studies focusing on other stakeholders are limited [7, 12]. To strengthen the dental therapy profession and improve healthcare delivery, it is essential to conduct targeted research that incorporates managerial insights and addresses structural barriers. This will inform effective HRH strategies for attracting, retaining and supporting dental therapists, ultimately enhancing equitable access to quality healthcare across South Africa.

Therefore, this study aimed to explore the perspectives of key stakeholders (regulators, provincial managers, academics and professional associations) on the factors contributing to attrition among dental therapists in South Africa given their influences on training, deployment and policy implementation. The scope of the study included stakeholders from five provinces and national organizations, with a specific focus on examining intrinsic and extrinsic factors as outlined in Herzberg's two‐factor theory [13] to identify the locus of specific action points.

## 2 Methodology

### 2.1 Study Design

This study utilized an exploratory qualitative design which was guided by the Hertzberg's two‐factor theory. This study adhered to the consolidated criteria for reporting qualitative research (COREQ) 32‐item checklist to promote transparent reporting of qualitative research [14].

### 2.2 Study Setting

The study was conducted in five out of nine provinces of South Africa where ethical clearance was obtained and participants gave consent to participate; these include the Eastern Cape, Free State, KwaZulu‐Natal, Northern Cape and North‐West. Furthermore, some participants, such as the representatives from Universities, the Health Professions Council of South Africa (HPCSA) and the South African Dental Therapy Association (SADTA), were interviewed who had jobs with a national scope.

### 2.3 Study Participants

Inclusion criteria required participants to have decision‐making, regulatory or academic roles involving dental therapy. Stakeholders without professional interaction with dental therapists were excluded. The participants included the provincial oral health managers/coordinators, dental therapy programme managers/coordinators from the two universities that train dental therapists (UKZN and SMU), a representative from SADTA as well as a representative from the HPCSA Board of Dental Therapy, Oral Hygiene and Dental Assisting. Participants were identified through provincial directories and university contacts and selected on the basis of their alignment with the inclusion criteria.

### 2.4 Sampling and Recruitment of Participants

Purposive sampling was employed to recruit participants from 1 March 2020 to 30 June 2022. We contacted and invited potential participants to join the study, and all oral health stakeholders who consented were included.

### 2.5 Data Collection

A semi‐structured interview schedule was developed for data collection. The interview guide included questions related to the role of dental therapists in the health system and the stakeholders’ perspective on the attrition of dental therapists. The interview guide was informed by existing literature and Herzberg's theory and reviewed by three senior co‐authors. The same interview guide was used across participants but adjusted slightly for role relevance. The interview guide was not formally piloted but was reviewed and refined by three senior co‐authors to ensure clarity and relevance prior to use. The three senior authors (S.J., V.Y. and S.N.) reviewed the data collection tool for content validity and clarity before data collection.

Stakeholders who consented to participate were allowed to be interviewed in person (at their workplace) or virtually (on Zoom or Teams). As a female dental therapist by training, MPH and PhD candidate, and who now works as an academic with experience in qualitative research, the first author (P.P.S.) conducted the semi‐structured in‐depth interviews (IDIs) to explore the stakeholders’ perspectives on attrition of dental therapists.

P.P.S. had no relationship with the participants before the study. The interviews were conducted in English and the took between 25 and 40 min. All interviews were audio recorded with participants’ consent. Interviews were conducted either face‐to‐face or virtually depending on the participant's preferences. P.P.S. also took detailed field noted during the IDIs which recorded the occurrences, discussions, observed behaviours, as well as her reflections on these interactions. Data collection was concluded after 12 interviews, as no new themes were emerging, indicating that data saturation had been reached.

### 2.6 Data Analysis

An independent transcriber transcribed the interviews verbatim; thereafter P.P.S. cross‐referenced the audio recordings with the transcripts for data accuracy. The transcripts were checked with members prior to analysis. Then, three authors (P.P.S., Y.M.‐K. and A.M.) conducted a thematic analysis, adhering to the method outlined by Braun and Clarke [15]. All transcripts were analysed using NVivo 12 qualitative analysis software. Given the first author's prior role as a dental therapist, potential bias was mitigated by involving a multidisciplinary team, including individuals without dental backgrounds, in analysis and interpretation. The team, comprising P.P.S., Y.M.‐K. (a dental specialist), A.M. (an oral hygienist) and S.J. (a non‐dental health professional), reviewed the transcripts for familiarization and insights. The initial codes were then developed by the team to form the first codebook which was further expanded until no new codes emerged, and the team felt comprehensive coverage of interview ideas. Trends, themes and sub‐themes were developed by merging codes and aligning similarities and differences in the data. An integration of data assessed links between participant information and finalized collectively in a meeting where the entire team was involved. To guarantee dependability, the last author, S.J., cross‐verified the final analysis, collaboratively resolving any discrepancies. The credibility of the study is found in the use of well‐established research methodology, and it is repeatable. The use of a wide range of stakeholders in different organization added the element of triangulation to enhance credibility. Ultimately, all authors participated in reviewing the document thoroughly to ensure its completeness and accuracy.

## 3 Ethical Considerations

Ethical approval to conduct the study was obtained from the University of the Witwatersrand's Medical Human Research Ethics Committee (HREC), certificate #M190686. The study also received approval from the six provincial ethics committees (Eastern Cape, Free State, Western Cape, Northern Cape and KwaZulu‐Natal), although we did not receive the consent to participate from one provincial manager; hence, only five provincial managers were included in the study. During the recruitment process, participants were reassured that the study was entirely voluntary, and their information would be kept confidential. The participants were informed that the information would be shared in scientific meetings and publications, and the research would be anonymous. On the day of the interview, P.P.S. obtained written informed consent from all respondents for participation in the study and for audio‐recording consent, which was signed and emailed before virtual interviews. To safeguard participants’ anonymity, codes were used instead of names.

## 4 Findings

A total of 12 stakeholders participated: nine individual interviews and one group discussion with three academics. These included five provincial oral health managers, two university dental therapy programme coordinators, one SADTA representative, one HPCSA board member and three academic staff (Table 1).

**TABLE 1:** Participants’ demographic characteristics.

Data collection method	Stakeholder	Gender
9 in‐depth interviews	5 Oral Health Provincial Managers	3 males
		2 females
	2 University programme managers	1 male
		1 female
	1 Dental therapy association (SADTA) representative	1 female
	1 HPCSA board member	1 male
1 small group discussion	3 Academics	3 females

Abbreviation: HPCSA, Health Professions Council of South Africa.

### 4.1 Themes Arising From the Data

Deductive themes structured a more inductive coding process. These included two broad themes: (1) dental therapist roles in the health system and (2) reasons for attrition. The reasons for attrition were deductively classified into extrinsic and intrinsic themes, derived from Herzberg's two‐factor theory [16]. The first theme, the role of dental therapists, pertains to the stakeholders’ comprehension of the role played by dental therapists within the South African health system. On the other hand, extrinsic and intrinsic themes were used to uncover the factors contributing to the attrition of dental therapists through the inductive coding process, through which the research team identified sub‐themes. The participating stakeholders directly articulated these themes and sub‐themes. Table 2 provides an overview of the primary (deductive) themes, two organizing themes and the associated sub‐themes identified through qualitative interviews.

**TABLE 2:** Themes arising from the data.

Theme		Sub‐theme	Description
**Role of dental therapists in the healthcare system**		Role in primary healthcare	When participants describe the role played by dental therapists in the healthcare system, especially in the primary healthcare setting
**Reasons for attrition**	Hygiene/Extrinsic factors	Gaps in policy implementation	When describing the exclusion of dental therapists in some human resource policies
		Poor remuneration	When describing low salaries for dental therapists in the public sector and low medical aid reimbursement in the private sector
		No recruitment	Description of recruitment of oral health professionals in the public sector
		No professional identity	Referring to group identity which refers to sense of belonging within the oral health team
	Motivators/Intrinsic factors	No career growth	When referring to growth within the dental therapy profession, advancing into managerial positions
		No career pathing	When referring to the postgraduate prospects of dental therapists

### 4.2 Theme 1: Role of Dental Therapists

Most stakeholders recognized the substantial and strategic role played by dental therapists within the South African health system, particularly at the primary healthcare (PHC) level. Their skills in prevention and basic restorative care align with the country's pressing oral health needs, especially in underserved regions. A provincial manager noted:

In our clinics, dental therapists are often the only oral health provider. Their role is crucial. (Provincial Manager 1)

This sentiment was echoed by stakeholders working directly with dental therapists, who emphasized their practical expertise and relevance to the current disease burden:

I think the therapists can make a huge difference because South Africa is very big, hey? And the disease profile is not getting any better. So, for us to be where we want to be as a country, to be able to control the oral health diseases. I think we need dental therapists. (Provincial Manager 2)

An Academic Coordinator further elaborated:

Dental therapists are trained with a strong preventive focus. This makes them essential in the PHC approach, but they are often overlooked in policy documents. (University A)

Several participants emphasized that the dental therapy scope is particularly well‐matched to the population's oral health needs.

A majority of our population needs basic dentistry, which is what the therapist does. So, that should be important to note, yeah. (Provincial Manager 3)

Recruitment practices also emerged as a concern. Some stakeholders suggested that dental therapists should be prioritized for permanent posts in the public sector:

If I were to be in charge, I would employ more therapists permanently in the government sector and let the dentists come for community service. But for permanent posts, I would recommend that we get more dental therapists. (Regulator 1)

The potential for dental therapists to contribute to broader health system reforms was also discussed, particularly in relation to the National Health Insurance (NHI). A regulator stated:

Dental therapists are very critical to our health care in South Africa and with the introduction of the National Health Insurance System, it becomes even more critical because remember that the NHI is aimed at primary health care. (Regulator 2)

However, perspectives were not universally informed. A provincial manager highlighted a gap in awareness and integration at the policy and oversight levels:

Remember, I am not in the dental profession. I got to take over seeing, but I'm not actually looking at every individual post… I wouldn't be really specific about it, because I'm not in service delivery level, I am in policing and strategic level. (Provincial Manager 4)

This diversity of perspectives illustrates a widespread consensus on the value of dental therapists, while also revealing a need for better integration and recognition of their role across all levels of health governance.

### 4.3 Theme 2: Reasons for Attrition

#### 4.3.1 Extrinsic Themes

##### 4.3.1.1 Gaps in Policy Implementation

Although South Africa has introduced several policies intended to facilitate the career advancement of health professionals, including dental therapists, stakeholders emphasized that implementation remains inconsistent across provinces and poorly enforced at the district level. This disconnects between policy design and practice has created a significant vacuum in the policy environment surrounding the dental therapy profession.

A provincial manager articulated this concern clearly:

Now, where a province can create posts at the Assistant Director and Deputy Director level for people doing clinical jobs, there will be an advantage. But then if you are in a province where such posts happen because of how the dispensation makes provision for that, it's up to the provinces to create such posts. If the provinces did not create such posts, then it means they won't be in a position to go up to that level. (Provincial Manager 3)

Another provincial manager added:

We've tried to implement the OSD posts, but without national enforcement, provinces vary widely. Some don't prioritize dental therapists at all. (Provincial Manager 1)

This lack of policy enforcement has a cascading effect on workforce planning and professional motivation. A manager described similar challenges in implementing human resource (HR) development plans at district level:

Much as our Head Office has forwarded a detailed training plan, there is still a serious challenge in the implementation of the Human Resource Development policies at the district level that would allow them to do advanced courses such as Simple Endo or Plastic dentures. These are essential for career pathways, including oral health management. (Provincial Manager 3)

The absence of structured postgraduate training opportunities was further echoed by academic representatives. An academic coordinator noted:

Our graduates often ask about postgrad options. But unless they shift to public health or education, there's no formal pathway. It's a dead end. (University A)

Collectively, these responses illustrate that although policies like the OSD aim to elevate dental therapists to senior clinical and managerial roles, their discretionary implementation by provinces undermines equitable career progression. This inconsistency erodes morale, limits professional growth and contributes directly to attrition.

##### 4.3.1.2 Poor Remuneration

Stakeholders consistently identified poor remuneration as a critical factor contributing to attrition among dental therapists. This issue was particularly evident in both the private and public sectors, where disparities between dental therapists and dentists were pronounced despite overlapping clinical responsibilities.

In the private sector, stakeholders emphasized inequities in reimbursement rates from medical aid schemes. A regulator highlighted:

Even in private sector, they will highlight an issue of saying we are performing the same duties, buying the same material but the codes—you find that sometimes it is 35% less if not 30% less. For the same restoration that the dental therapist would perform and the one that the dentist performs, the payments are not the same from the medical aid. (Regulator 1)

This concern was echoed by an academic, who stated:

This unequal valuation undermines their professional worth. If the market pays less for the same service, it sends a message that therapists are lesser. (University B)

From a policy standpoint, a regulator rationalized the pay gap by referencing differences in academic training duration:

When they explain the differentiation between the professions, they take into consideration the years trained. Like BDS is five years and dental therapy is three years, so they say remuneration can't be the same … even if the duties are similar. (Regulator 2)

In the public sector, participants noted that overtime remuneration policies are not designed with dental therapists in mind. Although dentists qualify for commuted overtime rates, dental therapists are assigned to general staff overtime categories. A regulator observed:

Commuted overtime is a document developed in the bargaining council, and it is for medical officers and dentists … those rates are higher than the one of the ordinary overtime, in a nutshell. (Regulator 2)

Another regulator added:

We do overtime and sometimes see more patients than the dentists. But when you look at the remuneration, it's lesser, it's not commensurate with the workload or skill. (Regulator 1)

These findings indicate a systemic undervaluation of dental therapists’ contributions, both in terms of market‐based fee structures and institutional compensation frameworks. This perceived inequity, particularly in light of workload and responsibilities, contributes significantly to job dissatisfaction and attrition.

##### 4.3.1.3 No Recruitment for Dental Therapists

Stakeholders widely acknowledged that dental therapists possess the clinical competencies to perform the majority of routine dental procedures typically undertaken in public healthcare settings. Their expertise in preventive care and basic treatment positions them as highly efficient providers, capable of meeting up to 95% of the oral health needs within the PHC framework.

As one academic coordinator stated:

The ideal person for primary and preventative health care would be your therapist … more than 90% of oral health‐related work is within the ambit of the dental therapist. It's your basic fillings, cleaning, extractions. They play an absolutely critical role. (University B)

Despite this recognized value, the recruitment of dental therapists into the public sector remains alarmingly limited. A manager noted that institutional hiring priorities are currently skewed towards absorbing community service dentists, leaving dental therapists, many of whom are bursary beneficiaries, without clear employment pathways:

There is little to nothing in terms of jobs, especially at district level. The focus is mainly on community service dentists who later become absorbed into permanent positions. The employment of DT/OH/DA is a big challenge, even though they are bursary holders. (Provincial Manager 3)

This gap between workforce supply and government planning has direct consequences for new graduates. An academic coordinator reflected on a recent year when no dental therapy graduates were employed in the public sector:

Last year's graduates, none of them had any posts in the public sector. The private sector had to absorb 40 to 50 therapists, which is appalling. There's a clear need, just no provision made for them. (University B)

An additional layer of concern was raised by one manager, who explained the unintended shift of the profession into private practice due to a lack of state employment:

Initially, therapy was meant for public service. But because there weren't enough jobs, they had to allow them into private practice. Personally, I think that was a mistake. The public sector needed them, and infrastructure was lacking. (Provincial Manager 5)

The downstream effect of poor recruitment practices is attrition through career‐switching. Even highly motivated dental therapists are compelled to abandon the profession in the absence of meaningful employment opportunities. A provincial manager remarked:

Those who want to be therapists cannot progress because there're no posts. Maybe they'll get my post when I resign. In a class of twenty, only one will succeed. The rest go back to school or turn to other professions. (Provincial Manager 1)

This lack of structured recruitment pathways, particularly within provinces where employment has dwindled to isolated cases, signals a misalignment between HRH planning and actual service delivery needs. A provincial manager confirmed:

In our province, we only have one dental therapist employed in the public sector. That's the extent of our recruitment. (Provincial Manager 3)

These insights underscore a critical failure in integrating dental therapists into the public sector health workforce, despite evident demand and established competencies. Addressing this issue is essential to achieving equitable healthcare access and optimizing the use of mid‐level oral health professionals.

##### 4.3.1.4 No Professional Identity

A recurring concern among stakeholders was the lack of professional identity of dental therapists, which contributes significantly to job dissatisfaction and attrition. Despite being legally recognized as autonomous practitioners with a defined scope of practice, dental therapists often face limited visibility and acknowledgment within both administrative structures and clinical teams.

A provincial manager candidly described the lack of recognition:

I don't think even people know who they are. I don't even think people know what a dental therapist is, what is his scope of practice, what is the difference between a dental therapist and a dentist. And what is it that they can do for us. No one knows about them, but they really could add value if they were known. I think maybe other provinces are handling it better. I am talking about my province. (Provincial Manager 5)

This lack of awareness at managerial levels perpetuates institutional neglect and hinders integration into the broader oral health workforce. From the perspective of frontline practitioners, the disconnect between scope of practice and supervisory expectations is a major demotivator. A regulator explained:

Even though our scope allows us to extract teeth and do restorations, many managers still think we need supervision for everything. It's frustrating. (Regulator 1)

The perception of exclusion extends to decision‐making and team dynamics. According to one regulator:

Dental therapists are sometimes excluded from dental team decisions. This affects morale and identity. (Regulator 1)

These insights reveal a profession caught in an identity crisis, formally trained and regulated, yet marginalized in practice. The misalignment between statutory recognition and organizational implementation not only diminishes morale but also reinforces professional invisibility.

The issue of identity is further compounded by the absence of strategic advocacy and underrepresentation in academic and regulatory forums. This lack of visibility impedes role modelling for students and limits upward mobility into leadership positions.

Collectively, these challenges underscore the urgent need for systemic efforts to bolster the professional identity of dental therapists. Such efforts should include awareness campaigns, organizational sensitization and leadership development initiatives aimed at integrating dental therapists more fully into the oral health workforce.

#### 4.3.2 Intrinsic Themes

##### 4.3.2.1 No Career Growth

A dominant intrinsic concern raised by stakeholders was the limited career growth opportunities for dental therapists within the public healthcare system. Despite their ability to manage service delivery, dental therapists are often not considered for senior or managerial positions, a reality that directly impacts professional motivation and long‐term retention.

A provincial manager acknowledged the contradiction:

They [dental therapists] will always run the dental clinic when there is no dentist, which is unfair. (Provincial Manager 4)

Despite managing key operations in the absence of dentists, dental therapists remain institutionally excluded from career advancement pathways:

You will never be, you work hard, but you will never be recognised to be a senior manager because of your scope of practice. You are always junior [laughs]. So, I think they have to work on that because there is no value placed on an individual unless they become a dentist. (Provincial Manager 4)

This perception, that dental therapists are inherently junior professionals, was echoed by others, who pointed to a narrow vision of what qualifies an individual for leadership roles. The prevailing belief appears to be that managerial eligibility is reserved for those with a full dental degree.

Dental therapists can be in a position to upgrade to the next level if they can get a little bit of a postgrad to be upgraded to a dentist. I think that will make it even much better. (Provincial Manager 4)

These statements reflect a structural limitation within the health system's HR frameworks, where career progression for dental therapists is either undefined or contingent upon transitioning out of the profession entirely. This dynamic not only discourages professional commitment but also suggests a systemic undervaluation of mid‐level providers as long‐term contributors to healthcare leadership.

The lack of defined clinical or administrative career ladders, combined with an institutional bias favouring dentists, reinforces the perception that dental therapy is a static or transitional career, rather than one with autonomous progression. This undermines both individual motivation and the system's ability to retain skilled personnel in the long term.

##### 4.3.2.2 No Career Paths

Stakeholders unanimously recognized the absence of structured career pathways for dental therapists as a significant barrier to professional growth and retention. Unlike their counterparts in dentistry, dental therapists currently lack defined postgraduate or specialized training routes that would enable vertical mobility within their field.

Responsibility for addressing this gap, according to stakeholders, lies with both the HPCSA and academic institutions. A regulatory Stakeholder emphasized this dual mandate:

One, I know that in terms of career pathing, HPCSA has the role to regulate the scopes, that is one. And universities are there to create the space or the environment for career pathing to be practical. (Regulator 2)

This suggests that although HPCSA defines the scope of professional competencies, universities are expected to operationalize these scopes through curriculum development and advanced training opportunities. However, current academic offerings remain limited, and dental therapists are often left without viable postgraduate options unless they transition into unrelated disciplines such as public health or education.

One manager criticized the narrow focus of career development initiatives in the public sector, pointing out that professional advancement tends to be disproportionately cantered on dentists:

This is one of the weaknesses in the public sector, to facilitate or prepare the opportunities for the Dental Therapy Profession to grow, other than for the dentists only. (Provincial Manager 5)

The absence of formal career ladders not only hinders the professional growth of dental therapists but also contributes to their marginalization within the health system. Without clear progression tracks, the profession risks being perceived as stagnant, thereby reducing its appeal to prospective students and increasing attrition among current practitioners.

To address this, stakeholders suggested the urgent need for collaborative frameworks between HPCSA, universities and provincial health departments to develop specialized diplomas, management training and clinical advancement programmes that align with the realities of public health delivery in South Africa.

## 5 Discussion

This study set out to explore key stakeholder perspectives on dental therapist attrition in South Africa, guided by Herzberg's two‐factor theory [16], in order to identify areas for action. The findings specified both extrinsic (policy implementation gaps, poor remuneration, limited recruitment and weak professional identity) and intrinsic (restricted career growth and lack of career pathways) factors that are shaping workforce attrition among dental therapists. These findings validate previous studies [7, 17, 18] that also demonstrate that the sustainability of the dental therapy workforce is undermined by both systemic and motivational challenges. What this study adds are managerial perspectives on the value of dental therapists what can be done to support them in the South African context.

The important role played by dental therapists in the health system emerged as a consistent area of stakeholder agreement. Participants described dental therapists as strategically positioned to deliver essential primary oral health services, particularly in under‐resourced communities. Their competencies in prevention, basic restorative care and patient education are well‐aligned to the country's oral health needs [18, 19, 20]. These views support global evidence suggesting that mid‐level oral health workers can address large‐scale treatment needs effectively and cost‐efficiently [1, 19]. Several stakeholders specifically linked the dental therapist role to the vision of South Africa's NHI, which is grounded in a PHC approach [21]. However, although support for the dental therapist role was robust, there was also evidence of uneven awareness, especially among higher level managers outside the dental profession. This indicates a need for more comprehensive stakeholder education to strengthen the professional recognition of dental therapists within oral health teams and policy frameworks [3].

The findings also highlight persistent extrinsic factors undermining retention, beginning with gaps in policy implementation. Although policies like the OSD and career development frameworks exist on paper, they are applied inconsistently across provinces. This weak enforcement, compounded by variable district‐level planning, leaves many dental therapists without opportunities to advance into senior or managerial roles. Similar patterns of policy–practice gaps have been observed across the South African health system more broadly [7], reflecting weaknesses in intergovernmental coordination. These policy gaps were repeatedly cited by participants as disempowering, stalling morale and contributing directly to professional exits.

Poor remuneration also emerged as a prominent extrinsic factor. Stakeholders described a systemic undervaluation of dental therapists’ contributions, with lower pay structures compared to dentists despite comparable tasks and responsibilities. In the private sector, medical aid reimbursement discrepancies reinforce a perception of dental therapists as ‘lesser’, which threatens their professional identity and retention [3, 19]. In the public sector, exclusion from commuted overtime schemes further limits fair compensation. These inequities appear to contradict both the workload realities of dental therapists and their scope of practice as autonomous mid‐level providers [4]. To date, there have been no significant national interventions beyond the Occupational Specific Dispensation to systematically address the compensation disparities faced by dental therapists, leaving the current inequities largely uncorrected. Closing these pay gaps will require a deliberate realignment of salary structures and reimbursement codes to match demonstrated competencies rather than purely years of academic training.

Recruitment bottlenecks represent another structural barrier. Despite clear evidence of their relevance to PHC needs, many dental therapists cannot find posts in the public sector after graduation. Stakeholders described a mismatch between community service recruitment pipelines (which favour dentists) and the practical needs for routine oral healthcare at PHC level. This results in underemployment or movement into the private sector, where remuneration challenges persist. Such failures of workforce planning reflect wider patterns in South African HR strategies, where mid‐level cadres are not systematically integrated into staffing models. Ensuring adequate recruitment of dental therapists requires explicit HRH policies that define their role, set minimum provincial post allocations and secure funding within PHC budgets.

Additionally, the lack of professional identity was a recurring theme, impacting both extrinsic and intrinsic motivation. Participants described how dental therapists remain poorly understood even within health teams, with scope‐of‐practice uncertainties, role confusion and a lack of voice in decision‐making structures. This undermines their sense of belonging and professional pride, reducing morale and encouraging attrition [3, 22]. Prior studies in other health professions have similarly shown that a weak professional identity is linked to low retention [23]. Strategic interventions to strengthen group identity, through advocacy campaigns, professional networking and leadership development, are critical to improving dental therapists’ standing in both policy and practice circles.

On the intrinsic (motivator) side, the study revealed deep frustration with the lack of career growth opportunities. Dental therapists, despite often managing clinics in the absence of dentists, remain blocked from senior positions due to narrow HR frameworks and a structural bias towards dentists for leadership roles. This results in a static career experience that many perceive as a ‘dead end’, a pattern widely recognized to demotivate health professionals [2, 24]. The lack of career pathways, including postgraduate specialization or advanced diplomas within dental therapy, forces some to switch careers entirely if they wish to advance, a finding consistent with global literature on mid‐level provider attrition [1].

Implications for policy and practice can be understood along a continuum of short‐ and long‐term interventions, as aligned with Herzberg's framework, which highlights the need to address both hygiene and motivator factors [16, 24]. In the short term, improvements can be made by enforcing consistent implementation of existing policies such as the OSD, prioritizing the creation of posts for dental therapists within provincial HRH plans and addressing recruitment bottlenecks. Targeted awareness campaigns and interprofessional collaboration to raise the visibility and professional identity of dental therapists are also feasible short‐term actions. In the longer term, more structural reforms are needed. These include revising remuneration frameworks to align compensation with a scope of practice, developing structured postgraduate training and specialization pathways and creating leadership development programmes. Such long‐term strategies will not only improve job satisfaction but also support the sustainable integration of dental therapists into South Africa's PHC vision.

These efforts will require strong advocacy and coordination between policymakers, regulatory bodies, academic institutions and professional associations. With committed leadership, these interventions can support both immediate workforce needs and the long‐term sustainability of the dental therapy profession. Future research should build on this stakeholder perspective by exploring dental therapists’ lived experiences directly, as well as testing interventions to address recruitment, remuneration and career pathing challenges. Evaluations of dental therapists’ integration into the NHI framework would also be valuable to inform South Africa's broader PHC reforms.

Limitations of the study should be noted, including a sample drawn from five provinces which may not fully represent national perspectives, and the focus on stakeholders rather than frontline dental therapists themselves, who need to be part of any reform initiatives. Nonetheless, the diverse voices included here provide a strong starting point to guide reforms.

The findings emphasizes that dental therapists are vital to realizing universal oral health coverage in South Africa. However, their continued marginalization, both structurally and professionally, threatens this potential. Coordinated policy, education and advocacy interventions are urgently needed to enable dental therapists to thrive and to deliver on South Africa's PHC promise.

## 6 Conclusion

This study highlights that dental therapists are indispensable to South Africa's PHC system, yet they face persistent barriers that undermine retention and professional satisfaction. To strengthen the dental therapy profession and improve healthcare delivery, it is essential to conduct targeted research that incorporates managerial insights and addresses structural barriers, as their suggestions reflect contextual insights into policy and practice that are otherwise inaccessible.

On the basis of their contributions, targeted interventions should include enforcing consistent policy implementation, revising remuneration and overtime frameworks, establishing structured recruitment pathways and creating opportunities for leadership and postgraduate training. Strengthening professional identity through advocacy and awareness campaigns is also critical to enhancing recognition and morale. Addressing these challenges will inform effective HRH strategies for attracting, retaining and supporting dental therapists, ultimately enhancing equitable access to quality healthcare across South Africa.

Future research should incorporate the voices of dental therapists themselves, evaluate the impact of retention strategies and examine their integration within NHI frameworks. Programmatic investments informed by these insights can optimize the dental therapy workforce, advance equity in oral health and contribute to South Africa's broader universal health coverage goals.

## Author Contributions

**Pumla Pamella Sodo**: conceptualization, data curation, formal analysis, investigation, methodology, project administration, validation, visualization, writing – original draft, writing – review and editing. **Yolanda Malele‐Kolisa**: formal analysis, writing – original draft, writing – review and editing. **Tshakane Ralephenya**: formal analysis, writing – review and editing. **Aneesa Moolla**: formal analysis, writing – review and editing. **Veerasamy Yengopal**: supervision, writing – review and editing. **Simon Nemutandani**: supervision, writing – review and editing. **Sara Jewett**: formal analysis, methodology, supervision, writing – review and editing.

## Conflicts of Interest

The authors declare no conflicts of interest.

## Data Availability

The qualitative interview data, which comprise of in‐depth interviews with 14 participants, cannot be shared openly. The Human Research Ethics Committee (HREC—Medical) of the University of Witwatersrand has imposed this restriction due to the small number of participants that could be identified easily, possibly compromising participant confidentiality and revealing sensitive information. For further clarification and requests, please contact the HREC chairperson, Professor P. Ruff through the secretariat at 011 717 2700/1234/2656/1252 and the e‐mail addresses are Zanele.Ndlovu@wits.ac.za, Rhulani.Mukansi@wits.ac.za, Mapula.Ramaila@wits.ac.za and Iain.Burns@wits.ac.za.
